# A chain mediation model on organizational support and turnover intention among healthcare workers in Guangdong province, China

**DOI:** 10.3389/fpubh.2024.1391036

**Published:** 2024-06-07

**Authors:** Yuanyuan Chen, Ping Xia, Chaojie Liu, Chumin Ye, Qi Zeng, Baofang Liang

**Affiliations:** ^1^School of Public Health and Management, Guangzhou University of Chinese Medicine, Guangzhou, China; ^2^School of Public Health, Guangdong Pharmaceutical University, Guangzhou, China; ^3^Centre for Research on Health Economics and Health Promotion, Guangdong Pharmaceutical University, Guangzhou, China; ^4^School of Psychology and Public Health, La Trobe University, Melbourne, VIC, Australia; ^5^Maoming Maternal and Child Health Hospital, Maoming, China

**Keywords:** turnover intention, organizational support, work-family-self balance, job satisfaction, healthcare workers

## Abstract

**Introduction:**

The outbreak of the Coronavirus Disease 2019 pandemic has presented significant difficulties for healthcare workers worldwide, resulting in a higher tendency to quit their jobs. This study aims to investigate the correlation between organizational support, work-family-self balance, job satisfaction, and turnover intention of healthcare professionals in China’s public hospitals.

**Methods:**

A cross-sectional survey was conducted on 5,434 health workers recruited from 15 public hospitals in Foshan municipality in China’s Guangdong province. The survey was measured by organizational support, work-family-self balance, job satisfaction, and turnover intention using a five-point Likert scale. The association between organizational support, work-family-self balance, job satisfaction, and turnover intention was investigated using Pearson correlation analysis and mediation analysis through the PROCESS macro (Model 6).

**Results:**

Organizational support indirectly affected turnover intention through three pathways: the mediating role of work-family-self balance, job satisfaction, and the chain mediating role of both work-family-self balance and job satisfaction.

**Conclusion:**

Health administrators and relevant government sectors should provide sufficient organizational support, enhance work-family-self balance and job satisfaction among healthcare workers, and consequently reduce their turnover intentions.

## 1 Introduction

The worldwide healthcare workforce has been enduring labor shortages, exacerbated by several factors, including heavy workloads, unpredictable work environments, the possibility of infection, the emotional toll of witnessing suffering and death, and the psychological strain stemming from the widespread COVID−19 pandemic (1, 2). These issues may lead to the dissatisfaction of healthcare workers and increase their likelihood of leaving their jobs (3). Turnover intention refers to the possibility that an employee willingly leaves his or her job in the future, which is a crucial predictor of turnover behavior (4, 5). Prior studies found that healthcare workers’ pooled prevalence of intention to leave was 30.4% in China (6) and 83% in Ethiopia (7). Several studies indicated that turnover intention among healthcare workers was high in Qatar (8) and Singapore (9) during the COVID−19 pandemic. However, a high turnover intention rate is not conducive to the continuity of patient care and the development of the healthcare system (8, 10).

Studies showed that different categories of healthcare workers had different intentions to leave. Global systematic reviews reported that general practitioners had a 47% prevalence of turnover intention (11), whereas pharmacists ranged from 13% to 61.2% (12). Other studies demonstrated that 43% of advanced practice nurses (13) and 39% of midwives had considered quitting their jobs (14). Additionally, 35 of 43 studies that examined healthcare workers’ turnover intention during the COVID-19 pandemic were focused on nurses, according to a global systematic review (15).

Turnover intention is affected by complex factors, including personal, organizational, and external environmental factors (16, 17). To examine the intrinsic motivation of turnover intention, various motivational models of turnover intention were proposed. The representative models are the Mobley model (18), the Steers and Mowday model (19), and the Price-Mueller model (20). One of the most influential models of employee turnover intention is the Price-Muller model. Based on expectancy theory, the model assumes that employees with a work environment that meets their values will be more satisfied, committed, and less likely to quit (21, 22). Furthermore, Price contends that when employees’ dissatisfactions exceed satisfactions, it puts them in a “costly” situation and drives them to look for other jobs (20). The model divides the factors influencing employee turnover into four categories: endogenous (e.g. job satisfaction and organizational commitment), structural (e.g. autonomy, pay and career), individual (e.g. training and affectivity), and environmental (e.g. opportunity and kinship responsibility).

To develop a more comprehensive turnover model, several studies have concentrated on identifying potential moderators of the association between organizational support and turnover intentions. Research indicates that organizational commitment and job satisfaction. mediated most associations between organizational support and turnover intention. A study identified that affective commitment acted as a mediating factor in the negative correlation between organizational support and turnover intention (23). Organizational concern and support may help healthcare workers foster their sense of belonging, leading to a high affective commitment and mitigating their turnover intention (23). Another study on 341 nurses revealed that job satisfaction mediated the negative relationship between organizational support and turnover intention (24). Healthcare workers who perceive sufficient support from their organizations are likely to work more effectively and efficiently, which increases job satisfaction and, in turn, decreases their intention to leave (25, 26).

Existing studies have explored the antecedents of healthcare workers’ intention to leave their jobs, mainly focusing on job characteristics, organizational variables, and personal factors, but much less is known about work-family-self balance. Moreover, the role of work-family-self balance in the relationship between organizational support and turnover intention of healthcare workers is little understood, and studies explaining the mechanism underlying the association between organizational support and turnover intention among healthcare workers were inadequate. In addition, during the COVID-19 epidemic, many studies of turnover intention were conducted on nurses rather than all healthcare workers. Therefore, this study aims to investigate the association between organizational support, work-family-self balance, job satisfaction, and turnover intention among healthcare workers in 15 public hospitals in Nanhai district, Foshan municipality, Guangdong province, China. We also aim to determine whether there is a connection between work-family-self balance and job satisfaction when both are considered to be the mediating role.

### 1.1 Literature review and hypothesis development

#### 1.1.1 Organizational support and turnover intention

Organizational support is typically assessed through a subjective perspective, capturing employees’ perceptions of how much the organization cares about their well-being and values their contributions (27). Sufficient organizational support has the potential to increase the sense of self-identity, belonging, responsibility, and work engagement of medical staff, thereby improving the relationship with their organization and boosting their confidence and skills in providing care for patients (28–31). In addition, organizational support was found to be a predictor of turnover intention and to have a negative relationship with it in earlier research (32). While low levels of organizational support result in higher turnover intention, employees who felt that their organizations provided adequate support could maximally mitigate their desire to leave and encourage them to stay (23, 33). Therefore, we propose the first hypothesis:

*H1*: Organizational support will be negatively associated with turnover intention.

#### 1.1.2 Work-family-self balance, organizational support, and turnover intention

“Satisfaction and good functioning at work and home, with a minimum of role conflict” was the term used to describe work-family balance (34). Work-life balance was defined as the ability of employees to work and fulfill their responsibilities to family and others outside of work (35). Work-family-self balance is the state of harmony among work, family, and self, involving physical and mental health (36). Previous studies showed that the COVID-19 pandemic increased healthcare workers’ workload and working hours, leading to longer shifts, disturbed sleep patterns, and fewer social and family activities (37, 38). According to the spillover and crossover theories, healthcare workers’ negative experiences, feelings, and attitudes at work would carry over into their homes and personal lives (39, 40). Research indicated that work-family balance had an impact on psychological outcomes, such as job anxiety, strain, and turnover intention (41, 42). A recent study found that four out of 10 healthcare workers did not balance their work with their non-work and community roles, negatively impacting their sense of personal and professional belonging (43) and potentially increasing turnover intention. However, the spillover effects and the crossover effects indicated that healthcare workers who felt they had adequate organizational support, including instrumental and emotional support (44), will get more stimulated, inspired, and have a positive experience at work, which they will carry over and transfer to their family and personal lives (39, 40). For example, they will interact with their families with greater joy, enthusiasm, and confidence (45), contributing to a more harmonious status among healthcare workers’ work and family, thereby reducing the likelihood of leaving their jobs. Existing research demonstrated that organizational support had a significant and positive effect on the work-life balance, that work-family balance and work-life balance were negatively associated with turnover intention, and that work-life balance may play a mediating role of organizational support and turnover intention (46). Based on these findings, we propose the second hypothesis:

*H2*: Work-family-self balance mediates the relationship between organizational support and turnover intention.

#### 1.1.3 Job satisfaction, organizational support, and turnover intention

Job satisfaction refers to an individual’s feelings about her/his job (47), and it is concerned with individual productivity, relationships with coworkers, physical and mental health, and life satisfaction (48). Furthermore, the quality of healthcare is influenced by the job satisfaction of healthcare workers (49). High job satisfaction may lead to increased productivity and creativity among healthcare personnel (50). In contrast, job dissatisfaction may result in adverse effects, including turnover and absenteeism, increased work accidents, and impairment to one’s mental and physical health (51). Additionally, job satisfaction was the most significant antecedent variable for predicting turnover intention (52). The extant research suggested that organizational support could increase job satisfaction and improve employees’ positive attitudes at work (53). Strongly perceived organizational support would result in high emotional connection and commitment to the organization (54), suggesting that employees are more likely to be content with their jobs rather than leave them, according to social exchange theory (25). In other words, organizational support would lower the intention to leave by improving job satisfaction (24). Therefore, we propose the third hypothesis:

*H3*: Job satisfaction mediates the relationship between organizational support and turnover intention.

#### 1.1.4 Work-family-self balance, job satisfaction, organizational support, and turnover intention

Based on the above theories, work-family-self balance and job satisfaction may mediate the correlation between organizational support and turnover intention of healthcare workers. Previous studies found that work-family balance and work-life balance were linked to increased job satisfaction and organizational performance (55–59). Work-family-self balance may have an initial impact on the association between organizational support and turnover intention, followed by job satisfaction. Then, we propose the fourth hypothesis:

*H4*: Work-family-self balance and job satisfaction play a chain mediating role in the relationship between organizational support and turnover intention.

## 2 Materials and methods

### 2.1 Study design and participants

A cross-sectional survey of hospital employees was conducted from 10 October 2021 to 11 January 2022. Study participants were recruited from 15 public hospitals (two primary hospitals with 20–99 beds, six secondary hospitals with 100–499 beds, and seven tertiary hospitals with ≥500 beds) in Nanhai District of Foshan municipality, Guangdong province, China. Eligible participants were contract staff of all professions on duty during the survey. Employees on leave at the time of the survey, retired personnel, and casual staff were not included.

A stratified proportional to size sampling method was used to recruit study participants. In Nanhai, the tertiary hospitals employed 6,877 professional healthcare workers, while the secondary hospitals employed 5,234 and the primary hospitals employed 572. Registered nurses made up about 49.68% of the skilled health workforce in Foshan. We set a quota for each participating hospital to recruit at least 220 participants. The sample size was estimated based on a sample size estimation for one-sample means $n={\left(\frac{Z_{1-\alpha /2}\sigma }{\delta }\right)}^{2}$ with α = 0.05, Z_1-α/2_ = 1.96, estimate of σ = 1.31, allowed error δ = 0.05, and 20% non-response rate was considered. The values of σ were derived from the results of the Fourth National Health Service Survey in Guangdong Province (60). Each professional group received a quota for each participating hospital based on size.

### 2.2 Data collection

The survey was performed via an online platform. Each hospital provided a demographic list of healthcare professionals, which was used to conduct stratified sampling based on three levels: profession, professional title, and gender. The phone numbers of the selected healthcare staff were provided by each hospital’s personnel department. The chosen healthcare workers were invited to complete the questionnaire, along with the respondent’s informed consent and a link to the questionnaire. Participation in the survey was voluntary and anonymous. A total of 5,702 questionnaires were distributed, and 5,434 (95.30%) of them were completed: 31.65% from physicians (*n* = 1,720), 45.29% from registered nurses (*n* = 2,461), 7.71% from pharmacists (*n* = 419), 8.52% from allied health workers (*n* = 463), and 6.83% from administrative staff (*n* = 371).

Respondents were invited to self-complete a questionnaire that included six close-ended questions about study participants’ sociodemographic and job characteristics and 32 close-ended questions for measuring organizational support, work-family-self balance, job satisfaction, and turnover intention. On average, each survey took about 4.27 minutes. We excluded incomplete and non-conforming questionnaires for quality control (less than 60 s of response time) (61).

### 2.3 Measures

#### 2.3.1 Organizational support

Organizational support was measured by a 5-item scale developed by Eisenberger et al. (6 items) (62). Each item was rated on a five-point Likert scale, with 1 representing “strongly disagree” and 5 representing “strongly agree.” The Cronbach’s alpha for organizational support scale was 0.980 in this study (Table 1). A summed score was calculated, with a higher score indicating higher levels of organizational support.

**Table 1:** The Cronbach’s alpha and KMO value of organizational support, work-family-self balance, job satisfaction, and turnover intention (*N* = 5,434).

Measure	Cronbach’s alpha	KMO
Organizational support	0.980	0.912
Work-family-self balance	0.971	0.873
Job satisfaction	0.987	0.978
Turnover intention	0.642	0.595

#### 2.3.2 Work-family-self balance

Work-family-self balance was measured by a 4-item scale developed by Xiao and Luo (4 items) (36). Each item was rated on a five-point Likert scale, with 1 representing “strongly disagree” and 5 representing “strongly agree.” The Cronbach’s alpha for work-family-self balance scale was 0.971 in this study (Table 1). A summed score was calculated, with a higher score indicating higher levels of work-family-self balance.

#### 2.3.3 Job satisfaction

Job satisfaction was measured using an 18-item scale developed by Zhang and Gu (18 items) (63). Respondents rated each item on a five-point Likert scale, with 1 representing “strongly disagree” and 5 representing “strongly agree.” The Cronbach’s alpha and Split-half reliability for job satisfaction scale was 0.987 and 0.973 (Table 1). A summed score was calculated for data analysis, with a higher score indicating higher levels of job satisfaction.

#### 2.3.4 Turnover intention

Turnover intention was measured by a 5-item scale adapted from the scale (items 32, 33, 34) developed by Mobley et al. (64) and the scale (items 35, 36) developed by Cammann (65). Each item was rated on a five-point Likert scale, with 1 representing “strongly disagree” and 5 representing “strongly agree.” The Cronbach’s alpha for turnover intention scale was 0.642 in this study (Table 1). A summed score was calculated, with a higher score indicating higher levels of turnover intention.

#### 2.3.5 Covariates

The covariates of this study included sociodemographic (gender, educational attainment) and job characteristics (profession, contract, professional title, position, and hospital grade). Empirical evidence shows that these characteristics are associated with job satisfaction and turnover intention (66, 67).

### 2.4 Statistical analysis

Data were entered into Excel and analyzed with SPSS 25.0 for Windows and PROCESS v4.0 macro (model 6) developed by Hayes (68). Respondents’ sociodemographic and job characteristics were described using frequency distributions for categorical and ordinal variables and mean values and standard deviations for continuous variables. Pearson correlation analysis was used to investigate the correlations between organizational support, work-family-self balance, job satisfaction, and turnover intention. Regression models were established to determine the mediating effect of work-family-self balance and job satisfaction on the association between organizational support and turnover intention. Four models were established: model one tested the effect of organizational support on turnover intention; model two tested the impact of organizational support on work-family-self balance; model three tested the mediating effect of work-family-self balance on the association between organization support and job satisfaction; model four examined the mediating effects of work-family-self balance and job satisfaction on the correlation between organizational support and turnover intention, as well as the chain mediating effect of work-family-self balance and job satisfaction on the relationship between organizational support and turnover intention. Seven covariates (gender, academic qualification, work experience, profession, professional title, leadership position, and hospital grade) were controlled in all models. Indirect effects were computed using a bias-corrected bootstrapping procedure. If the 95% confidence interval (CI) did not include zero, it indicated that the mediation effect was significant. Missing values were handled using a pairwise deletion approach. A two-sided *p*-value of less than 0.05 was considered statistically significant.

## 3 Results

### 3.1 Characteristics of study participants

The vast majority were female (70.61%), do not have a postgraduate qualification (92.58%), held a middle professional title (40.93%), and were not in a leadership position (85.11%). Most worked in a tertiary hospital (58.15%) and had over 10 years of work experience (52.87%). Registered nurses comprised 45.29% of the study population, followed by physicians (31.65%; Table 2).

**Table 2:** Socio-demographic characteristics of healthcare staff in the Nanhai district (*N* = 5,434).

Characteristics	Category	*N*	%
Gender	Male	1,597	29.39
	Female	3,837	70.61
Qualification	Undergraduate or below	5,031	92.58
	Postgraduate	403	7.42
Years of work experience	<5	1,109	20.41
	5–10	1,440	26.50
	>10	2,873	52.87
	missing	12	0.22
Profession	Physician	1720	31.65
	Registered nurse	2,461	45.29
	Pharmacist	419	7.71
	Allied health worker	463	8.52
	Administrative staff	371	6.83
Professional title	None	237	4.36
	Junior	1912	35.19
	Middle	2,224	40.93
	Associate senior	847	15.59
	Senior	214	3.94
Leadership position	No	4,625	85.11
	Yes	809	14.89
Hospital grade	Primary	222	4.09
	Secondary	2052	37.76
	Tertiary	3,160	58.15

### 3.2 Organizational support, work-family-self balance, job satisfaction, and turnover intention

Respondents reported a mean score of 4.262 (SD = 0.907), 4.171(SD = 0.926), 4.363 (SD = 0.782), and 2.478 (SD = 0.806) for organizational support, work-family-self balance, job satisfaction, and turnover intention, respectively. Organizational support, work-family-self balance, job satisfaction, and turnover intention were all moderately or strongly correlated, with correlation coefficients ranging from −0.454 to 0.927 (Table 3).

**Table 3:** Descriptive statistic and Pearson correlations among organizational support, work-family-self balance, job satisfaction, and turnover intention.

Variables	Mean ± SD	Organizational support	Work-family-self balance	Job satisfaction	Turnover intention
Organizational support	4.262 ± 0.907	1.000	–	–	–
Work-family-self balance	4.171 ± 0.926	0.846**	−1.000	–	–
Job satisfaction	4.363 ± 0.782	0.927**	0.835**	1.000	–
Turnover intention	2.478 ± 0.806	−0.451**	−0.454**	−0.441**	1.000

SD, Standard Deviation; **p < 0.01(two-tailed).

### 3.3 Results of regression modeling and mediating effects

Respondents with higher organizational support (*β* = −0.413, *p* < 0.001) had lower levels of turnover intention (Model 1 in Table 4). Hypothesis 1 is confirmed. Respondents with higher organizational support (*β* = 0.867, *p* < 0.001) had higher levels of work-family-self balance (Model 2 in Table 4). Respondents with higher organizational support (*β* = 0.669, *p* < 0.001) and higher work-family-self balance (*β* = 0.151, *p* < 0.001) had higher levels of job satisfaction (Model 3 in Table 4). Respondents with higher organizational support (*β* = −0.167, *p* < 0.001), higher work-family-self balance (*β* = −0.203, *p* < 0.001), and higher job satisfaction (*β* = −0.087, *p* < 0.05) had lower levels of turnover intention (Model 4 in Table 4).

**Table 4:** Regression analysis of the relationship between organizational support and turnover intention (*N*=5,422).

**Regression equation**		**Fitting index**			**Significance**	
**Dependent variable**	**Predictor variable**	**R**	**R²**	***F***	***β***	***t***
Turnover intention	Organizational Support	0.461	0.213	182.879***	−0.413	−37.693***
Work-family-self balance	Organizational Support	0.846	0.716	1708.727***	0.867	114.966***
Job satisfaction	Organizational Support	0.934	0.872	4107.369***	0.669	84.286***
	Work-family-self balance				0.151	19.578***
Turnover intention	Organizational Support	0.481	0.231	162.663***	−0.167	−5.476***
	Work-family-self balance				−0.203	−10.061***
	Job satisfaction				−0.087	−2.525*

*p<0.05; **p<0.01; ***p<0.001(two-tailed).

Work-family-self balance partially mediated the relationship between organizational support and turnover intention: the indirect effect accounted for 42.62% of the total effect. Job satisfaction partially mediated the relationship between organizational support and turnover intention, with the indirect effect accounting for 14.04% of the total effect. According to the chain mediation that described the mediating effects of work-family-self balance and job satisfaction between organizational support and turnover intention, the indirect impact contributed to 2.66% of the total effect (Table 5). Hypotheses 2, 3, and 4 are confirmed.

**Table 5:** Work-family-self balance and job satisfaction in the mediation effect analysis.

Paths	Effects	Boot SE	Boot LLCI	Boot ULCI	Relative mediation effect
Total effect	−0.413	0.011	−0.434	−0.391	—
Direct effect	−0.167	0.031	−0.227	−0.107	—
Total indirect effect	−0.246	0.026	−0.296	−0.193	59.56%
Indirect path 1	−0.176	0.016	−0.207	−0.145	42.62%
Indirect path 2	−0.058	0.022	−0.101	−0.014	14.04%
Indirect path 3	−0.011	0.005	−0.020	−0.003	2.66%

Indirect path 1: the indirect effect of organizational support on turnover intention via work-family-self balance. Indirect path 2: the indirect effect of organizational support on turnover intention via job satisfaction. Indirect path 3: the indirect effect of organizational support on turnover intention via work-family-self balance and job satisfaction. LLCL, Lower Limit of Confidence Interval; ULCL, Upper Limit of Confidence Interval.

**Figure 1:**
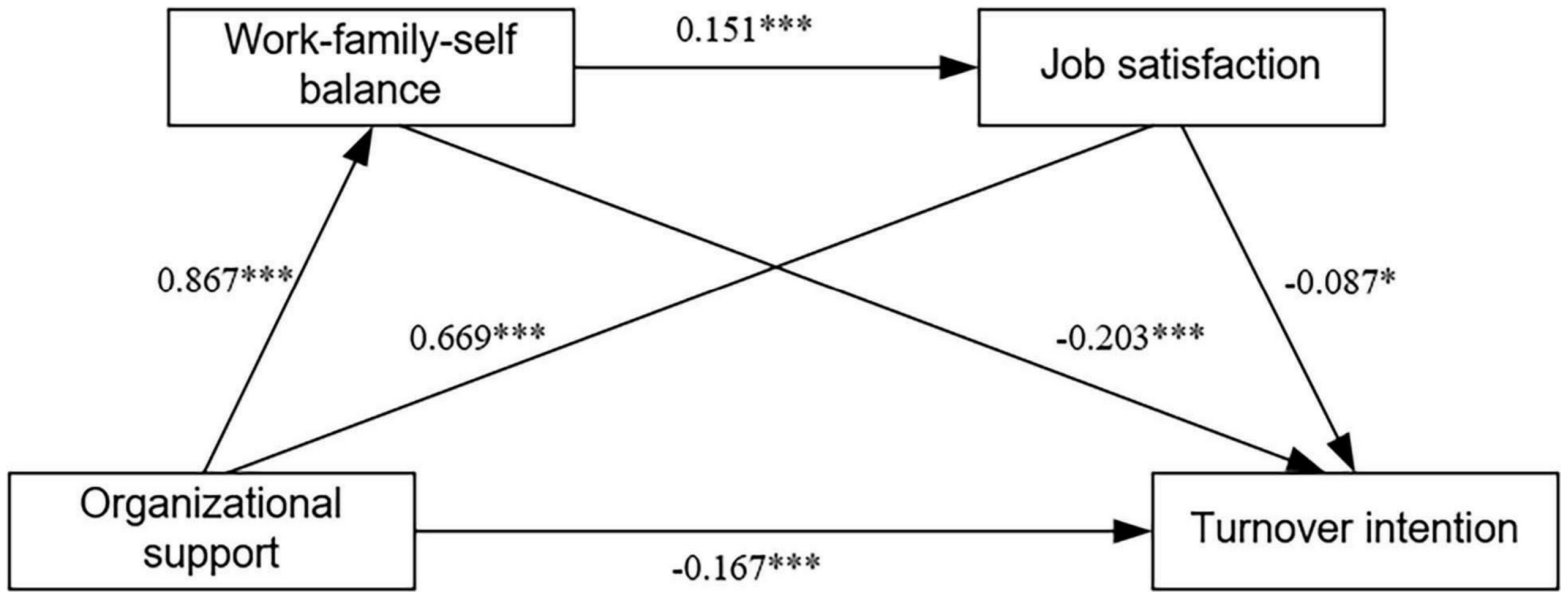
The chain mediation model. The chain mediating role of organizational support and turnover intention through work-family-self balance and job satisfaction. *p < 0.05; **p < 0.01; ***p < 0.001(two-tailed).

Figure 1 illustrates how organizational support can indirectly predict turnover intention via the single mediating effect of work-family-self balance and job satisfaction and the chain mediating effect of work-family-self balance and job satisfaction. The mediating effect consists of three indirect effects: Path 1: organizational support → work-family-self balance → turnover intention. Path 2: organizational support → job satisfaction → turnover intention. Path 3: organizational support → work-family-self balance → job satisfaction → turnover intention. All three indirect impacts are significant.

## 4 Discussion

This study investigated the effect of organizational support on healthcare workers’ turnover intention by collecting data from healthcare workers in Guangdong province, China, during the COVID-19 pandemic. We explored the chain mediating effects of work-family-self balance and job satisfaction on organizational support and turnover intention among healthcare workers. This is the first study to examine the relationship between organizational support, work-family-self balance, job satisfaction, and turnover intention in China. Additionally, this study contributed to the theoretical development and applications of turnover intention.

The study found that organizational support, work-family-self balance, and job satisfaction had a direct impact on turnover intention, which is consistent with prior research. An earlier study suggested that certain supportive practices, such as participation in decision-making and job rewards, could help to reduce employee turnover intention (32). A study revealed that healthcare workers who struggled to balance their work and family lives were more likely to experience job anxiety, which led to a greater intention to leave their jobs (69). Another study of primary care doctors in China indicated that individuals under age 30 were more likely to consider leaving their positions in search of better career opportunities and being dissatisfied or negative about their current job prospects (70). Organizational support, work-family-self balance, and job satisfaction directly affect turnover intention since they are linked to an employee’s career and psychological well-being.

This current study found that the associations between organizational support and turnover intention among healthcare workers are mediated by work-family-self balance and job satisfaction, which is consistent with previous studies (46, 71). Furthermore, this study investigated a chain link between organizational support, work-family-self balance, job satisfaction, and turnover intention. These findings suggest that organizational support may contribute to work-family-self balance and job satisfaction and that work-family-self balance may contribute to job satisfaction. Healthcare workers who receive more organizational support may feel respected and valued by the organization, promoting positive emotions in the workplace, such as a sense of security and belonging (72). According to social exchange theory (25), when healthcare workers feel positive emotions and attitudes at work, their job satisfaction is considerably increased. Meanwhile, adequate organizational support is necessary for healthcare workers to balance the demands of their professional work, family responsibilities (45), and personal lives (73, 74) while also improving their mental health (75–78). Healthcare workers who are well-supported by their organizations are more productive, creative, and happy. Healthcare workers’ increased work productivity and creativity have the potential to alleviate the stress caused by heavy workloads and strict work hour pressures, reducing conflict between work, family, and themselves and facilitating the achievement of their desired state of work-family-self balance (69, 79). In addition, implementing work-life balance practices in hospitals could help healthcare workers maintain a balance between their professional and personal lives, enhancing their performance at work and increasing their job satisfaction (80). Organizational support directly affects work-family-self balance, job satisfaction, and turnover intention, as well as indirectly affects job satisfaction and turnover intention. This emphasizes the importance of organizational support in healthcare workers’ well-being and turnover decision-making, highlighting the need for organizations to adopt supportive measures. To improve organizational support for healthcare workers, creating a supportive work environment (81) and formulating organizational policies and human resource initiatives (82) may facilitate balancing work demands outside of the workplace, such as encouraging organizational trust and positive organizational climate (44), providing training and development opportunities, and establishing family supportive policies (27). Work-family-self balance also has a substantial impact on turnover intention, indicating that healthcare workers’ needs outside of work should be addressed and work-family-self-balance initiatives implemented. Providing benefits assists healthcare workers in achieving a better work-family-self balance and improving job performance by expanding health insurance coverage for healthcare workers and their dependents and introducing programs or services to promote their physical well-being and fitness (83).

The study explores the role of work-family-self balance and job satisfaction as the internal mechanisms that link organizational support to turnover intention among healthcare workers, thereby enriching the understanding of how organizational support influences turnover intention and emphasizing the importance of organizational support in mitigating turnover intention. The findings of this study provide a foundation for further research to understand the complexity and multidimensionality of healthcare workers’ turnover intention. Moreover, this study has important implications for health and hospital administrators, indicating that strengthening organizational support levels could facilitate achieving work-family self-balance, enhance job satisfaction, and lower turnover intention. The results of this study offer initial evidence that preventive intervention approaches should be prioritized to improve work-family selfbalance and job satisfaction among healthcare workers. There are some limitations in this study. First, it is not ideal that Cronbach’s coefficient of turnover intention scale was below 0.7. We will try to find ways to improve the reliability of the results in further research. Second, the sample size for the two primary hospitals did not reach 220 because of the small number of staff in the primary hospitals. Third, this study did not measure demographic characteristics of age. Finally, the generalization of this study’s findings needs to be tested and verified further, as all participants come from the same district.

## 5 Conclusion

The study showed that organizational support indirectly predicts the turnover intention of healthcare workers through the mediating effects of work-family-self balance, job satisfaction, and the chain mediating effect between work-family-self balance and job satisfaction. This suggests that health administrators and relevant government sectors should be reminded of the significance of providing organizational support. They should enhance healthcare workers’ work-family-self balance, boost their job satisfaction, and decrease their intention to leave.

## Data availability statement

The raw data supporting the conclusions of this article will be made available by the authors, without undue reservation.

## Ethics statement

The studies involving humans were approved by the Ethics Committee of Guangdong Hospital of Traditional Chinese Medicine. The studies were conducted in accordance with the local legislation and institutional requirements. Written informed consent for participation in this study was provided by the participants’ legal guardians/next of kin.

## Author contributions

YC: Formal analysis, Validation, Writing – original draft, Resources. PX: Conceptualization, Funding acquisition, Methodology, Project administration, Supervision, Writing – review & editing. CL: Methodology, Writing – review & editing, Conceptualization, Supervision. CY: Investigation, Writing – original draft, Resources, Data curation. QZ: Data curation, Investigation, Validation, Writing – original draft. BL: Data curation, Investigation, Validation, Writing – original draft.
