# Corrigendum: A chain mediation model on organizational support and turnover intention among healthcare workers in Guangdong province, China

**DOI:** 10.3389/fpubh.2024.1448762

**Published:** 2024-09-09

**Authors:** Yuanyuan Chen, Ping Xia, Chaojie Liu, Chumin Ye, Qi Zeng, Baofang Liang

**Affiliations:** ^1^School of Public Health and Management, Guangzhou University of Chinese Medicine, Guangzhou, China; ^2^School of Public Health, Guangdong Pharmaceutical University, Guangzhou, China; ^3^Centre for Research on Health Economics and Health Promotion, Guangdong Pharmaceutical University, Guangzhou, China; ^4^School of Psychology and Public Health, La Trobe University, Melbourne, VIC, Australia; ^5^Maoming Maternal and Child Health Hospital, Maoming, China

**Keywords:** turnover intention, organizational support, work-family-self balance, job satisfaction, healthcare workers

In the published article, there were three errors in Table 3 as published. The correlation coefficients of work-family-self balance and work-family-self balance is incorrectly written as −1.000. The correlation coefficients of job satisfaction and job satisfaction is incorrectly written as −1.000. The correlation coefficients of turnover intention and turnover intention is incorrectly written as −1.000. The corrected Table 3 and its caption “Descriptive statistic and Pearson correlations among organizational support, work-family-self balance, job satisfaction, and turnover intention” appear below.

**Table 3:** Descriptive statistic and Pearson correlations among organizational support, work-family-self balance, job satisfaction, and turnover intention.

**Variables**	**Mean ±SD**	**Organizational support**	**Work-family-self balance**	**Job satisfaction**	**Turnover intention**
Organizational support	4.262 ± 0.907	1.000	–	–	–
Work-family-self balance	4.171 ± 0.926	0.846^**^	1.000	–	–
Job satisfaction	4.363 ± 0.782	0.927^**^	0.835^**^	1.000	–
Turnover intention	2.478 ± 0.806	−0.451^**^	−0.454^**^	−0.441^**^	1.000

SD, standard deviation; ^**^p < 0.01(two-tailed).

In the published article, there was an error. The mistake was a word misuse.

A correction has been made to **Materials and method section**, *Study design and participants*, Paragraph 2. This sentence previously stated:

“… allowance error δ=0.05.”

The corrected sentence appears below:

“… allowed error δ = 0.05.”

The authors apologize for these errors and state that these do not change the scientific conclusions of the article in any way. The original article has been updated.

